# Female Sex as a Risk Factor for Ischemic Stroke and Systemic Embolism in Chinese Patients With Atrial Fibrillation: A Report From the China‐AF Study

**DOI:** 10.1161/JAHA.118.009391

**Published:** 2018-09-26

**Authors:** Di‐Hui Lan, Chao Jiang, Xin Du, Liu He, Xue‐Yuan Guo, Song Zuo, Shi‐Jun Xia, San‐Shuai Chang, Song‐Nan Wen, Jia‐Hui Wu, Yan‐Fei Ruan, De‐Yong Long, Ri‐Bo Tang, Rong‐Hui Yu, Cai‐Hua Sang, Rong Bai, Nian Liu, Chen‐Xi Jiang, Song‐Nan Li, Jian‐Zeng Dong, Gregory Y. H. Lip, Ai‐Hua Chen, Chang‐Sheng Ma

**Affiliations:** ^1^ Department of Cardiology Heart Center Zhu Jiang Hospital Southern Medical University Guangzhou China; ^2^ Department of Cardiology Beijing AnZhen Hospital Capital Medical University National Clinical Research Centre for Cardiovascular Diseases Beijing China; ^3^ Heart Health Research Center Beijing China; ^4^ Institute of Cardiovascular Sciences University of Birmingham United Kingdom

**Keywords:** atrial fibrillation, ischemic stroke, women, Atrial Fibrillation, Women, Ischemic Stroke

## Abstract

**Background:**

Previous studies have provided conflicting results as to whether women are at higher risk than men for thromboembolism in the setting of atrial fibrillation (AF). We investigated whether women with AF were at higher risk of ischemic stroke in the China‐AF (China Atrial Fibrillation Registry) Study.

**Methods and Results:**

A total of 19 515 patients were prospectively enrolled between August 2011 and December 2016 in the China‐AF Study. After exclusion of patients receiving anticoagulation or ablation therapy, 6239 patients (2574 women) with results from at least 6 months of follow‐up were used for the analysis. Cox proportional hazards models were performed to evaluate whether female sex was an independent risk factor for thromboembolism after multivariate adjustment. The primary outcome was the time to the first occurrence of ischemic stroke or systemic embolism. After a mean follow‐up of 2.81±1.46 years, 152 female patients reached the primary outcome, as compared with 172 male patients. Crude incidence rates of thromboembolism between women and men were of borderline statistical significance (2.08 versus 1.68 per 100 patient‐years, *P*=0.058). After multivariable analysis, female sex was not independently associated with an increased thromboembolism risk (hazard ratio 1.09, 95% confidence interval 0.86‐1.39). There was no significant difference in thromboembolism risk by sex stratified by age and presence or absence of risk factors (*P* for interaction all >0.1).

**Conclusions:**

Although crude incidence rates of thromboembolism were higher in Chinese female patients with AF compared with male patients, female sex did not emerge as an independent risk factor for thromboembolism on multivariate analysis.

**Clinical Trial Registration:**

URL: http://www.chictr.org.cn/. Unique identifier: ChiCTR‐OCH‐13003729.

**Clinical Perspective**

**What Is New?**

- Our study showed that female sex did not emerge as an independent risk factor for stroke and systemic embolism in Chinese atrial fibrillation patients.
- No significant interactions between sex and age or comorbidities was found for thromboembolism.

**What Are the Clinical Implications?**

- Our findings do not support consideration of oral anticoagulant treatment for female Chinese patients with a CHA_2_DS_2_‐VASc score of 1.
- Initial decisions on oral anticoagulant treatment guided by a CHA_2_DS_2_‐VA approach (ie, ignoring the Sex category [Sc] criterion) may be appropriate.

## Introduction

Atrial fibrillation (AF) is the most common sustained arrhythmia and is associated with a 5‐fold increase in the risk of ischemic stroke.^1^ Usually, female AF patients tend to be older, with more comorbidities, and therefore to have a higher incidence of stroke.^2^, ^3^ However, whether female sex is a prognostic factor for stroke is still uncertain. Large‐cohort studies have indicated that women with AF are at 10% to 90% higher risk of stroke than men,^2^, ^4^, ^5^, ^6^, ^7^, ^8^ whereas others failed to find such an association.^9^, ^10^, ^11^, ^12^ Notably, the ATRIA (Anticoagulation and Risk Factors in Atrial Fibrillation) study indicated that women were at 60% and 80% increased stroke risk than men for those aged ≤75 and >75 years respectively,^4^ whereas 3 other studies have reported that female sex is associated with 10% to 23% higher risk of stroke only for patients over the age of 75.^6^, ^7^, ^8^ Furthermore, the Danish nationwide cohort study suggested that the excess stroke risk for women was evident among those with ≥2 non–sex‐related risk factors.^13^ Conversely, a recent Korean nationwide cohort study showed that female patients had a lower risk of ischemic stroke than males.^14^ Hence, contemporary European and American AF guidelines are inconsistent in their recommendations as to whether female sex should be used as a risk factor in deciding anticoagulation therapy.^15^, ^16^

In this study we used data from the China‐AF (China Atrial Fibrillation Registry) cohort to evaluate whether women are at higher risk for stroke or systemic embolism (SE) after adjustment of other confounding factors and whether the association is consistent among different subgroups.

## Methods

For the concern about intellectual property and patient privacy, the data, analytic methods, and study materials of this study will not be made available to other researchers for purposes of reproducing the results or replicating the procedure.

### Study Population

**Figure 1:**
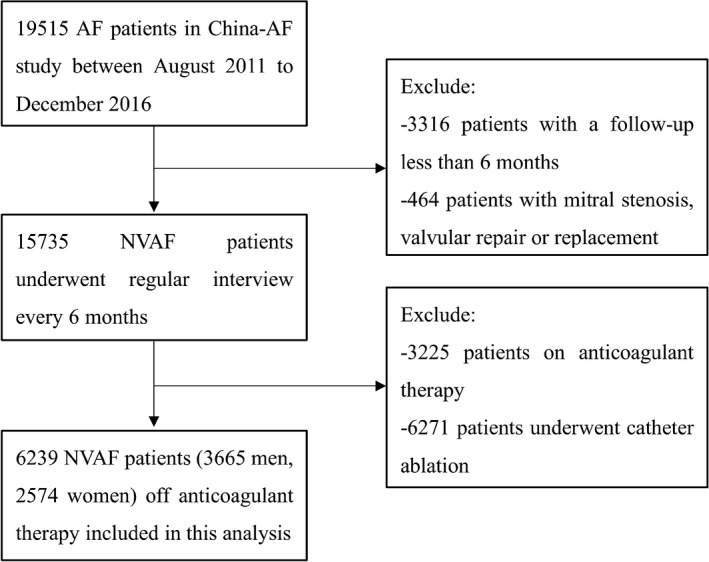
Flowchart of patients included. AF indicates atrial fibrillation; NVAF, nonvalvular atrial fibrillation.

The China‐AF study is a prospective, multicenter, hospital‐based, ongoing registry study of patients diagnosed with AF. The main purpose of this observational study is to understand the current clinical practice patterns, prognoses, and related factors in Chinese AF patients and to compare different treatments in real‐world practice. Details of the cohort have been described previously.^17^ Ethics approval was obtained from the local institutional review board, and written informed consent was obtained from patients. From August 2011 to December 2016, 19 515 AF patients were recruited from outpatient clinics and in‐hospital patients of 31 hospitals located in Beijing, China. Patients were followed up at 3 months, 6 months, and every 6 months thereafter. In this analysis we excluded patients with a follow‐up of less than 6 months (n=3316) and patients with mitral stenosis or valvular repair or replacement (n=464). Patients receiving oral anticoagulant treatment at baseline (n=3225) were excluded, and we also excluded patients who underwent catheter ablation (n=6271) to exclude any possible impact of the procedure on stroke risk.^18^, ^19^, ^20^ Finally, 6239 participants were included in this analysis. A flowchart of the study is shown in Figure 1.

Information on patient characteristics, including age, sex, lifestyle factors, type of AF, medical history, medication, education, and insurance, was collected when patients were enrolled. Definitions of each variable were in line with the American College of Cardiology/American Heart Association recommendation on AF clinical data standards and international peer studies.^21^

### Follow‐Up

Each enrolled patient was followed up at outpatient clinic or through telephone interview every 6 months. Information of medical or interventional therapies, events of incident ischemic stroke/SE, bleedings, hospitalizations, and deaths was collected at each follow‐up occasion. Person‐time was censored at the time the patient initiated oral anticoagulant therapy, catheter ablation was applied, first ischemic stroke/SE or death occurred, or the time data were analyzed.

### Ascertainment of Thromboembolic Events

The primary outcome was the time to the first occurrence of a thromboembolic event, including ischemic stroke and SE, whichever came first. Patient‐reported nonfatal thromboembolic events were adjudicated by 2 independent neurologists separately. Disagreement was resolved by discussion or by involving other senior neurologists.

### Definitions

The estimated glomerular filtration rate based on creatinine was calculated with the modified equation for Chinese patients with chronic kidney disease.^22^ The CHADS_2_ score was calculated for each patient by giving 1 point to each patient of age ≥75 years, history of hypertension, diabetes mellitus, and congestive heart failure and 2 points to each patient with a history of thromboembolism.^23^ We also calculated the CHA_2_DS_2_‐VASc score by giving 2 points to each patient of age ≥75 years and a history of thromboembolism and 1 point to each patient of age 65 to 74 years, history of hypertension, diabetes mellitus, congestive heart failure, vascular disease, and female sex.^24^ A sexless CHA_2_DS_2_‐VASc score (ie, removing female sex), abbreviated as CHA_2_DS_2_‐VA, was calculated by excluding female sex from CHA_2_DS_2_‐VASc score.

### Statistical Analyses

Descriptive statistics were used to compare demographic characteristics, comorbid conditions, and concomitant medication between men and women. Continuous variables were presented as mean and standard deviation or median and interquartile range as appropriate. A t test or the Wilcoxon rank‐sum test was used to compare the differences. Dichotomous variables were presented as percentages and were compared by χ^2^ test. Incidence rate of thromboembolic events was reported as the number of events per 100 person‐years of follow‐up in women and men, respectively. Cumulative incidence rates were estimated with the Kaplan‐Meier method and compared with the log‐rank test by sex and age groups.

Cox proportional hazards regression was employed to estimate the association between sex and stroke in a series of models with incremental adjustments as follows: model 1 was adjusted for age as a continuous variable; model 2 was adjusted for age, congestive heart failure, hypertension, diabetes mellitus, thromboembolism, and vascular disease; and model 3 was adjusted for all covariates with *P*<0.2 in the univariate Cox regression model. Results are expressed as hazard ratios with their 95% confidence intervals (CIs). Interactions between selected variables and sex were tested after multiple adjustment and presented in a forest plot. If covariate data were missing for the Cox regression models, PROC MIANALYZE was used, with a multiple imputation method, to calculate hazard ratios and their 95% CIs and to conduct valid statistical inferences from 5 imputed data sets.

All statistical analyses were performed using SAS version 9.4 (SAS Institute, Cary, NC). All statistical tests were 2‐sided; a *P*<0.05 was considered statistically significant.

## Results

We included 2574 women and 3665 men with AF in this analysis: of these, 2576 (41.29%) were identified during hospitalization, 3500 (56.10%) were enrolled from outpatient clinics, and 163 (2.61%) were enrolled from the emergency department. Baseline characteristics of male and female patients were shown in Table 1. Women were generally older (mean age 70.2 years versus 65.8 years, *P*<0.0001) and were more likely to have a history of heart failure, hypertension, or diabetes mellitus but were less likely to have diagnosed vascular disease than men. Women had higher mean CHADS_2_ (2.0 versus 1.7, *P*<0.0001) and CHA_2_DS_2_‐VASc (3.9 versus 2.4, *P*<0.0001) scores.

**Table 1:** Baseline Characteristics by Sex

Characteristics	Whole Cohort (N=6239)	Men (N=3665)	Women (N=2574)	*P* Value
Age, y	67.6±12.6	65.8±13.4	70.2±10.8	<0.0001
BMI, kg/m^2^	25.1±3.6	25.2±3.5	25.0±3.8	0.1324
SBP, mm Hg	129.7±17.8	128.6±16.9	131.3±18.8	<0.0001
Pulse pressure, mm Hg	51.9±15.3	50.2±14.6	54.2±15.9	<0.0001
Heart rate, bpm	81.7±21.9	80.8±20.9	82.9±23.2	0.0003
eGFR <60, mL/(min/1.73 m^2^)	347/4615 (7.5)	136/2640 (5.2)	211/1975 (10.7)	<0.0001
Echocardiography				
Anteroposterior left atrial diameter, mm	40.6±8.1	41.0±7.9	40.0±8.4	<0.0001
Moderate to severe mitral regurgitation	294/4488 (6.6)	160/2618 (6.1)	134/1870 (7.2)	0.1593
Smoking				
Current	974/6198 (15.7)	903/3639 (24.8)	71/2559 (2.8)	<0.0001
Former	1056/6198 (17.0)	963/3639 (26.5)	93/2559 (3.6)	
Never	4168/6198 (67.2)	1773/3639 (48.7)	2395/2559 (93.6)	
Alcohol use				
Current	1101/6193 (17.8)	1063/3635 (29.2)	38/2558 (1.5)	<0.0001
Former	718/6193 (11.6)	692/3635 (19.0)	26/2558 (1.0)	
Never	4374/6193 (70.6)	1880/3635 (51.7)	2494/2558 (97.5)	
AF type				
Newly diagnosed	852/6236 (13.7)	457/3664 (12.5)	395/2572 (15.4)	0.0006
Paroxysmal	3297/6236 (52.9)	1928/3664 (52.6)	1369/2572 (53.2)	
Persistent	2087/6236 (33.5)	1279/3664 (34.9)	808/2572 (31.4)	
Medical history				
Congestive heart failure	1492/6239 (23.9)	768/3665 (21)	724/2574 (28.1)	<0.0001
Hypertension	4177/6239 (66.9)	2278/3665 (62.2)	1899/2574 (73.8)	<0.0001
Diabetes mellitus	1618/6239 (25.9)	845/3665 (23.1)	773/2574 (30.0)	<0.0001
Thromboembolism	1027/6238 (16.5)	582/3664 (15.9)	445/2574 (17.3)	0.1410
Ischemic stroke	891/6238 (14.3)	505/3664 (13.8)	386/2574 (15.0)	0.1775
Vascular disease	1390/6238 (22.3)	868/3664 (23.7)	522/2574 (20.3)	0.0014
Previous bleeding	325/6237 (5.2)	199/3663 (5.4)	126/2574 (4.9)	0.3470
Hyperlipidemia	1875/6217 (30.2)	979/3652 (26.8)	896/2565 (34.9)	<0.0001
Hypertrophic cardiomyopathy	50/6226 (0.8)	26/3657 (0.7)	24/2569 (0.9)	0.3312
Dilated cardiomyopathy	53/6238 (0.8)	41/3664 (1.1)	12/2574 (0.5)	0.0057
Stroke risk score				
CHADS_2_	1.8±1.5	1.7±1.5	2.0±1.5	<0.0001
CHA_2_DS_2_‐VASc	3.0±2.0	2.4±1.9	3.9±1.9	<0.0001
Concomitant medication				
Antiplatelet	1674/6239 (26.8)	1010/3665 (27.6)	664/2574 (25.8)	0.1221
Statins	2596/6239 (41.6)	1437/3665 (39.2)	1159/2574 (45.0)	<0.0001
ACEIs/ARBs	2545/6238 (40.8)	1381/3664 (37.7)	1164/2574 (45.2)	<0.0001
Completed high school	1615/5496 (29.4)	1185/3252 (36.4)	430/2244 (19.2)	<0.0001
Health insurance coverage				
100%	795/6239 (12.7)	517/3665 (14.1)	278/2574 (10.8)	0.0006
Partially	4944/6239 (79.2)	2857/3665 (78.0)	2087/2574 (81.1)	
None	500/6239 (8.0)	291/3665 (7.9)	209/2574 (8.1)	

Data are shown as mean±SD or n/N (%). History of thromboembolism includes ischemic stroke, transient ischemic attack and systemic embolism. Alcohol use is defined as at least 20 g of pure alcohol on 1 occasion for both men and women. Previous bleeding means clinically relevant major or nonmajor bleeding. ACEIs indicates angiotensin‐converting enzyme inhibitors; AF, atrial fibrillation; ARBs, angiotensin II receptor blockers; BMI, body mass index; eGFR, estimated glomerular filtration rate; SBP, systolic blood pressure.

### Thromboembolic Events

During a mean follow‐up of 2.81±1.46 years, 324 thromboembolic events (291 ischemic strokes and 33 systemic embolisms) occurred. Women accrued 152 thromboembolic events, whereas men accumulated 172 events, with corresponding incidence rates of 2.08 and 1.68 per 100 patient‐years, respectively. Crude incidence rates of thromboembolism between women and men were of borderline statistical significance (*P*=0.058).

**Figure 2:**
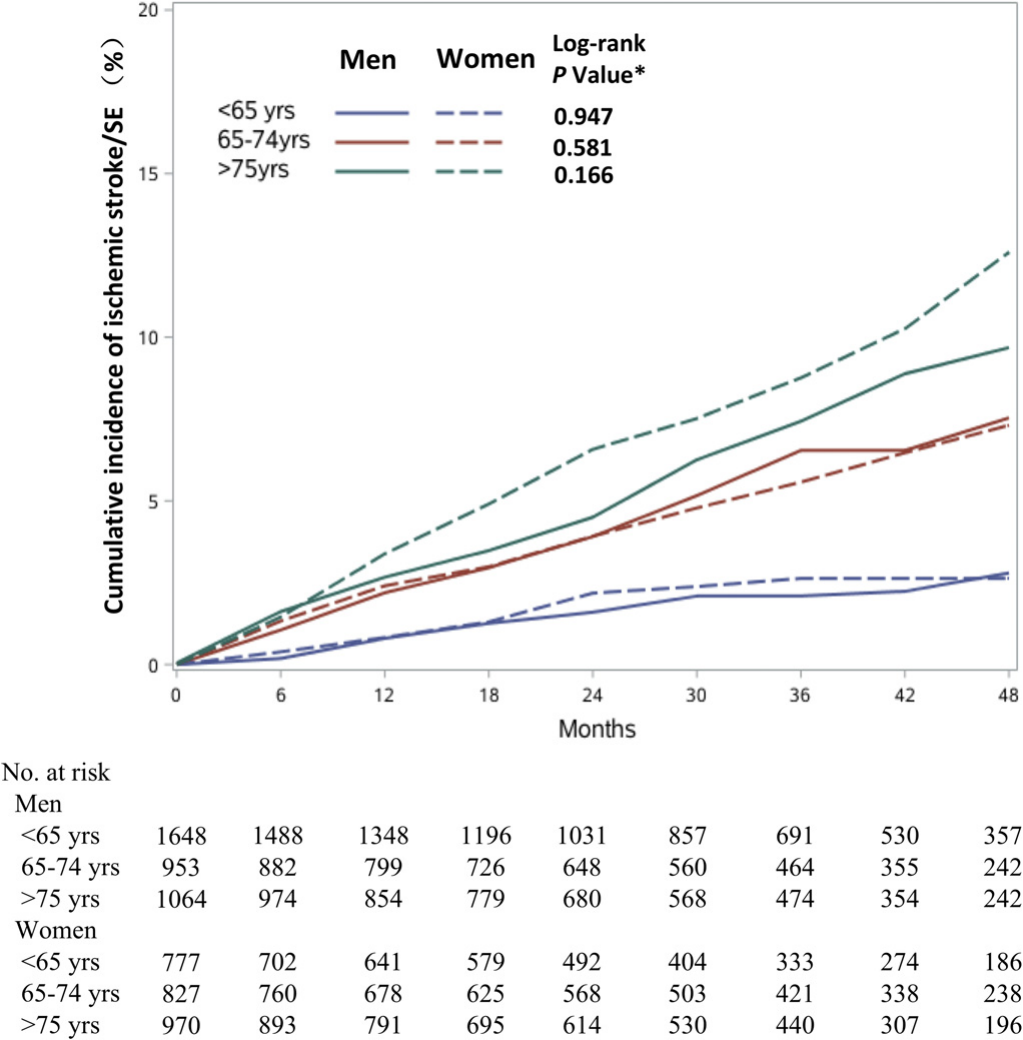
Cumulative incidence rates of ischemic stroke/SE in women and men, stratified by age groups. *Comparisons between men and women, by Log‐rank test. SE indicates systemic embolism.

Cumulative incidence rates of ischemic stroke/SE subdivided by sex and age groups (<65, 65‐74, and ≥75 years) are shown in Figure 2. On Kaplan‐Meier analysis, women were at not at higher risk of stroke/SE than similar‐aged men across different age groups.

**Figure 3:**
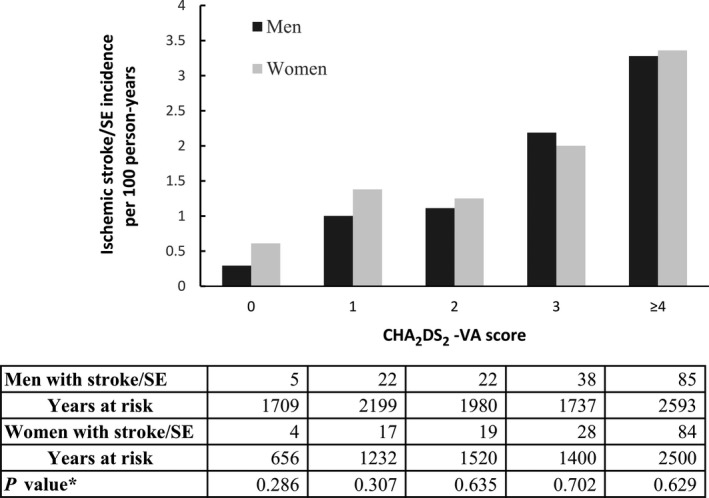
Crude incidence rates of ischemic stroke/SE per 100 person‐years by sex according to CHA_2_ DS_2_‐VA score. *Comparison between men and women by Fisher exact test. SE indicates systemic embolism.

Women had nonsignificantly different incidences of thromboembolism than men for each CHA_2_DS_2_‐VA score group (Figure 3). The absolute risk of thromboembolism was low among patients with a CHA_2_DS_2_‐VA score of 0, being 0.61, 0.29 per 100 patient‐year for women and men (*P*=0.286), respectively.

### Multivariate Analysis

After age had been adjusted as a continuous variable, female sex was still nonsignificantly associated with thromboembolic risk (hazard ratio 1.06, 95% CI 0.85‐1.32, Table 2). The association remained nonsignificant (hazard ratio 1.02, 95% CI 0.82‐1.27) even after adjustment for age, history of congestive heart failure, hypertension, diabetes mellitus, thromboembolism, and vascular disease. The results were similar (hazard ratio 1.09, 95% CI 0.86‐1.39) when all covariates with *P*<0.2 in univariate cox regression model (including age, estimated glomerular filtration rate, left atrial diameter, moderate‐to‐severe mitral regurgitation, history of congestive heart failure, hypertension, diabetes mellitus, thromboembolism, vascular disease, previous bleeding, use of angiotensin‐converting enzyme inhibitor and/or angiotensin II receptor blocker, and completion of high school) were adjusted. Table S1 provides all risk estimates of the different covariates included in the univariate and multivariate Cox regression model.

**Table 2:** Association Between Female Sex and Incidence of Stroke/SE

	Men	Women
Hazard ratio (unadjusted)	Reference	1.24 (0.99‐1.54)
Hazard ratio (adjusted for age^a^)	Reference	1.06 (0.85‐1.32)
Hazard ratio (adjusted for age^a^, history of heart failure, hypertension, diabetes mellitus, thromboembolism, vascular disease)	Reference	1.02 (0.82‐1.27)
Hazard ratio (adjusted for variables with *P*<0.2 in univariate Cox regression models)	Reference	1.09 (0.86‐1.39)

a Age considered as a continuous variable. SE indicates systemic embolism.

### Subgroup Analysis

**Figure 4:**
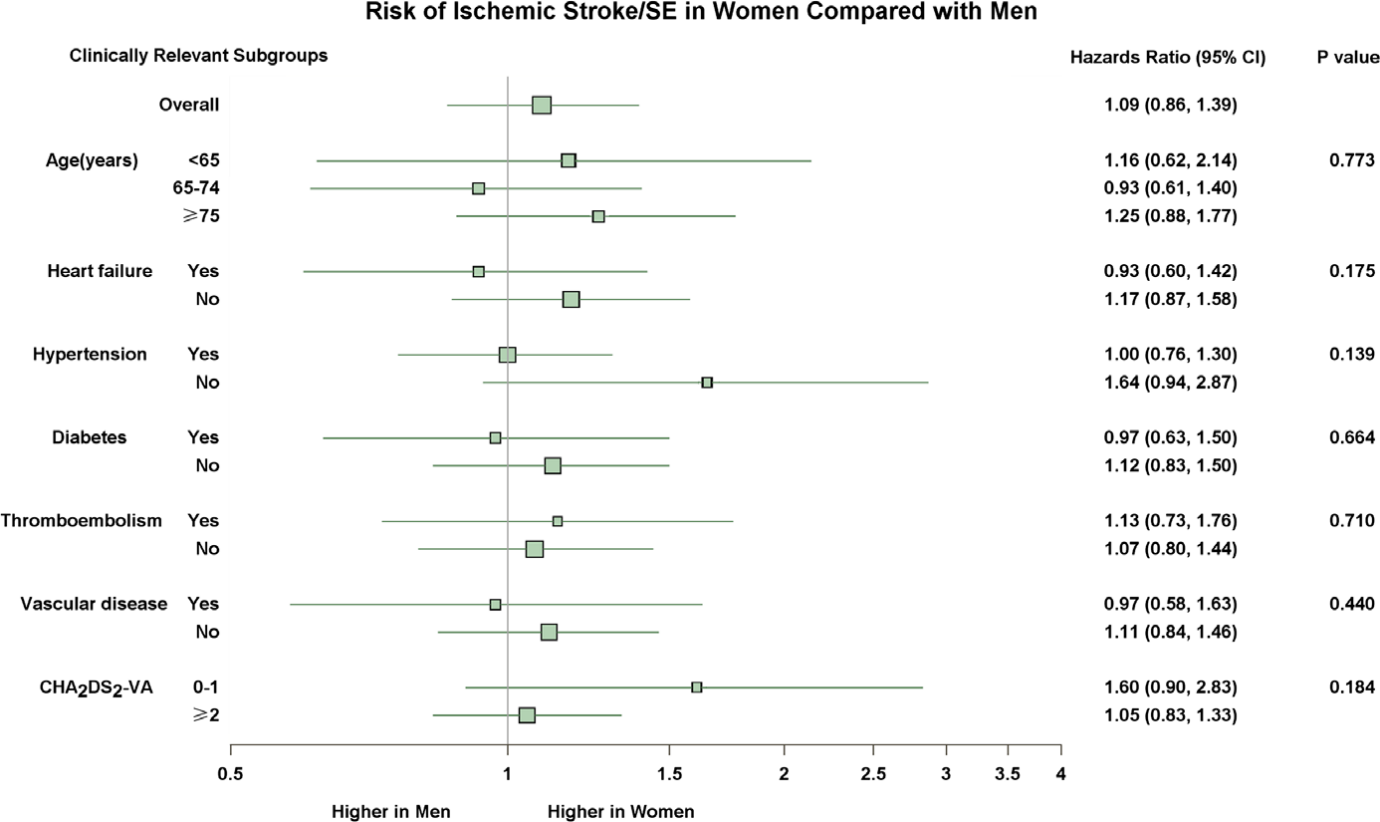
Risk of ischemic stroke/SE in women compared with men in clinically relevant subgroups. All interactions modeled within the previous multivariable Cox regression, except CHA_2_ DS_2_‐VA score (in which only sex and CHA_2_ DS_2_‐VA score were included in the model). SE indicates systemic embolism.

In subgroups of different age groups (<65, 65‐74, and ≥75 years), with and without history of heart failure, hypertension, diabetes mellitus, thromboembolism, as well as vascular diseases, with CHA_2_DS_2_‐VA score ≤1 or ≥2, we found no significant interactions between sex and clinically relevant variables (Figure 4).

## Discussion

In this large cohort of Chinese patients with AF, we found that although crude annual incidence rates of thromboembolism were higher in Chinese female patients with AF compared with male patients, female sex did not emerge as an independent risk factor for stroke and systemic embolism on multivariate analysis. In addition, no significant interactions between sex and age or comorbidities were found for thromboembolism.

The debate as to whether female sex is an independent risk factor for thromboembolic events in patients with AF remains. The earlier Framingham and ATRIA studies initially reported female sex as a risk factor for stroke.^4^, ^5^ However, these cohorts were conducted in the era of a relatively low awareness of AF and less optimal management of the arrhythmia and associated risk factors. The recognition of AF as a significant clinical condition and improvements in treatment of comorbidities may have contributed to the elimination of sex differences in stroke.

Three more recent large‐cohort studies in Canada, Sweden, and Denmark suggested that there was a significant interaction between age and sex for the risk of stroke. Female sex was a risk factor for stoke only in patients aged ≥75 years, not in those aged <75 years.^6^, ^7^, ^8^ However, the Canadian cohort revisited their data recently and found no significant association between female sex and risk of thromboembolism in patients older or younger than 75 years after matching on age and using time‐dependent adjustment for confounders.^9^ In addition, the Danish cohort also reviewed their data from 3 nationwide registries recently and showed that female sex had significant interaction with other risk factors and could be considered a risk modifier rather than a risk factor for stroke in patients with AF.^13^ Other analyses based on the UK General Practice Research Database and Asian cohort studies also found no sex differences in stroke risk.^10^, ^11^, ^14^, ^25^, ^26^

Of note, none of the registry‐based studies collected information about lifestyle factors such as smoking, alcohol, and obesity, which may affect the risk of thromboembolic events.^27^, ^28^, ^29^ The Danish Diet, Cancer and Health study suggested no sex difference in stroke risk after controlling for lifestyle‐related factors, including smoking, alcohol, obesity, and hormone replacement therapy.^12^ All of these findings suggest that possible unadjusted confounders may contribute to the observed association between female sex and risk of stroke. In our study additional adjustment for echocardiographic, lifestyle, and socioeconomic factors did not alter the association between female sex and stroke risk.

Our study indicates that female sex is not an independent risk factor for stroke in Chinese AF subjects and should not necessarily be incorporated into their risk stratification for guiding anticoagulation therapy decisions. Current European AF treatment recommendations are the same for female and male patients based on additional risk factors beyond sex.^15^ In contrast, American guidelines still provide the possibility of initiating anticoagulation based on female sex alone.^16^ However, our findings do not support consideration of oral anticoagulant treatment for female Chinese patients with a CHA_2_DS_2_‐VASc score of 1, given that these patients displayed an absolute risk of thromboembolism of 0.61 per 100 patient‐years.

Contemporary Canadian and Japanese AF guidelines also do not include female sex as a risk factor in considering anticoagulation treatment.^30^, ^31^ Indeed, an analysis from the J‐RHYTHM Registry indicated that the CHA_2_DS_2_‐VA score, a risk‐scoring system that excludes female sex from CHA_2_DS_2_‐VASc and performs better in risk stratification for thromboembolic events than the CHA_2_DS_2_‐VASc score, especially in identifying truly low‐risk Japanese AF patients.^32^ Nonetheless, the J‐RHYTHM registry recorded anticoagulation use only at baseline, and events at follow‐up may be confounded by anticoagulation use among high‐risk subjects.

Although our findings suggest that female AF patients are not exposed to extra risk of thromboembolic events, it is important to realize that AF was more commonly detected at first stroke in women and was associated with worse stroke outcomes.^33^ Women with AF are more likely to be underdiagnosed and managed conservatively. It is reported that women with AF are less likely to receive anticoagulation therapy.^34^ However, anticoagulant therapy is at least as beneficial in women as in men with AF.^35^ Specific attention is warranted to promote anticoagulation therapy among women with AF. Indeed, initial decisions on oral anticoagulant treatment could be guided by a CHA_2_DS_2_‐VA score (ie, excluding the sex category criterion), but the Sc risk component may modify and possibly increase stroke risk in women with ≥2 additional stroke risk factors.^13^

However, some limitations of the study should be noted. First, the patients in this study were derived from hospitals in Beijing; thus, more symptomatic or sicker patients were possibly more likely to be selected. Also, the numbers of patients and events in our prospective cohort were not comparable with the other nationwide registries. In addition, only information about patient characteristics taken at the baseline was used for the analyses, and hence, the effects of changes in increasing age and incident comorbidities were not taken into consideration.^36^, ^37^ AF categorization in our study did not differentiate permanent AF from persistent AF. Because this categorization applies to both male and female patients, the results are less likely to be impacted by this limitation. Finally, we lacked information about hormone replacement therapy, which may have a role in stroke risk; however, this potential bias is likely to be minimal because the awareness and use of hormone replacement therapy are comparatively low in China.^38^

## Conclusions

Crude incidence rates of thromboembolism were higher in Chinese female patients with AF compared with male patients, but female sex did not emerge as an independent risk factor for stroke and systemic embolism on multivariate analysis. In Chinese AF subjects, initial decisions on oral anticoagulant treatment guided by a CHA_2_DS_2_‐VA approach (ie, ignoring the Sex category [Sc] criterion) may be appropriate.

## Sources of Funding

This study was supported by a grant (2013BAI09B02) from the Ministry of Science and Technology of the People's Republic of China and grants (ZYLX201302, D111100003011004, and D131100002313001) from Beijing Municipal Commission of Science and Technology. The construction of the China‐AF was also supported by grants from Bristol‐Myers Squibb, Pfizer, Johnson & Johnson, Boehringer‐Ingelheim, and Bayer.

## Disclosures

Dr Ma received honoraria from Bristol‐Myers Squibb, Pfizer, Johnson & Johnson, Boehringer‐Ingelheim, and Bayer for delivering lectures. Dr Lip has served as a consultant for Bayer/Janssen, Bristol‐Myers Squibb/Pfizer, Boehringer Ingelheim, Novartis, Verseon, and Daiichi‐Sankyo and as a speaker for Bayer, Bristol‐Myers Squibb/Pfizer, Boehringer Ingelheim, and Daiichi‐Sankyo. No fees were directly received personally. Dr Dong also received honoraria from Johnson & Johnson for delivering lectures. The remaining authors have no disclosures to report.

## Supporting information


**Table S1.** Associations Between Baseline Factors and Ischemic Stroke/SE
