# Supplementary material for: Female Sex as a Risk Factor for Ischemic Stroke and Systemic Embolism in Chinese Patients With Atrial Fibrillation: A Report From the China‐AF Study
Source: J Am Heart Assoc. 2018 Sep 26;7(19):e009391. doi: 10.1161/JAHA.118.009391 (PMC6404890; doi:10.1161/JAHA.118.009391)
Supplement: Supplementary file 1 — Table S1. Associations Between Baseline Factors and Ischemic Stroke/SE [file JAH3-7-e009391-s001.pdf]

SUPPLEMENTAL MATERIAL

Table S1. Associations Between Baseline Factors and Ischemic Stroke/SE.

Variable	Univariate Analysis		Multivariate Analysis	
	HR (95%CI)	P value	HR (95%CI)	P value
Sex, female	1.24 (0.99-1.54)	0.058	1.09 (0.86-1.39)	0.482
Age, per year	1.05 (1.04-1.07)	<.0001	1.04 (1.02-1.05)	<.0001
BMI>28kg/m^2	0.83 (0.60-1.15)	0.265	/	/
SBP>140mmHg	1.42 (1.14-1.78)	0.002	/	/
Pulse pressure>60mmHg	1.31 (1.01-1.69)	0.041	/	/
Heart rate>100bpm	1.01 (0.74-1.38)	0.939	/	/
eGFR<60 mL/min/1.73m^2	1.42 (0.92-2.18)	0.110	0.69 (0.43-1.11)	0.129
Left atrial diameter>40mm	1.57 (1.21-2.04)	0.001	1.45 (1.06-2.00)	0.0225
Moderate-to-severe mitral regurgitation	1.44 (0.90-2.31)	0.127	1.07 (0.67-1.72)	0.780
Smoking (any)	0.87 (0.68-1.10)	0.240	/	/
Alcohol use (any)	0.86 (0.67-1.10)	0.220	/	/
Persistent AF	1.15 (0.92-1.44)	0.220	/	/
Medical history				
Heart failure	1.76 (1.39-2.22)	<.0001	1.15 (0.88-1.51)	0.312
Hypertension	1.98 (1.50-2.60)	<.0001	1.48 (1.07-2.04)	0.0168
Diabetes mellitus	1.46 (1.16-1.84)	0.002	1.16 (0.90-1.50)	0.248
Thromboembolism	2.51 (1.98-3.17)	<.0001	2.03 (1.57-2.64)	<.0001
Vascular disease	1.24 (0.96-1.59)	0.097	0.86 (0.65-1.14)	0.307
Previous bleeding	1.97 (1.35-2.86)	<.0001	1.25 (0.81-1.93)	0.308

Hyperlipidemia	1.08 (0.85-1.36)	0.540	/	/
Use of Statins	1.04 (0.84-1.30)	0.706	/	/
Use of ACEIs/ARBs	1.19 (0.95-1.48)	0.127	0.86 (0.67-1.10)	0.228
Completed high school	0.75 (0.57-0.98)	0.032	0.87 (0.66-1.16)	0.344
Health insurance (any)	1.22 (0.79-1.88)	0.363	/	/

BMI, body mass index; SBP, systolic blood pressure; eGFR, estimated glomerular filtration rate; ACEIs, angiotensin-converting enzyme inhibitors; ARBs, angiotensin II receptor blockers.
